# Investigating the physicochemical, rheological, and sensory properties of low‐fat mayonnaise prepared with amaranth protein as an egg yolk replacer

**DOI:** 10.1002/fsn3.4163

**Published:** 2024-04-10

**Authors:** Sahar Mohammadi, Mazdak Alimi, Seyed‐Ahmad Shahidi, Shirin Shokoohi

**Affiliations:** ^1^ Department of Food Science and Technology, Ayatollah Amoli Branch Islamic Azad University Amol Iran; ^2^ Chemical, Polymeric and Petrochemical Technology Development Research Division Research Institute of Petroleum Industry Tehran Iran

**Keywords:** amaranth protein, egg replacement, emulsion stability, mayonnaise, rheological properties

## Abstract

This study investigated the possibility of using amaranth protein isolate (API) as a plant‐based substitute for egg yolk (EY) in the preparation of low‐fat mayonnaise (LFM). The alkali extraction/acidic precipitation method was used to isolate amaranth protein; its functional properties were then studied. The results showed that besides its great water and oil absorption capacities, API had better emulsifying capacity and significantly higher (*p* < .05) emulsion stability at pH 2.0 than alkali pH values. Five mayonnaise samples with different API/EY combination ratios (%) (i.e., 0/0.75, 0.25/0.5, 0.375/0.375, 0.5/0.25, and 0.75/0) were prepared. The color, emulsion stability (ES), freeze–thaw stability (FTS), droplet size, structure, rheology, and sensory properties of samples were examined. API replacement showed no adverse effects on the L* value, ES, and sensory attributes (*p* > .05). Low API concentrations (0.25% and 0.375%) significantly (*p* < .05) increased the droplet size and decreased the FTS of LFM emulsion. High API concentrations (0.5% and 0.75%) had no significant effect (*p* > .05) on droplet size and formed emulsions with more tightly packed oil droplets. The Cross model was chosen best to describe the flow behavior of LFM samples (*R* ^2^ = 0.99). The sample with 0.75% API had significantly (*p* < .05) the highest values of *η*_*o*_ (zero‐shear viscosity) and *λ* (relaxation time), indicating greater interaction between the emulsion particles. All samples showed a weak gel structure (*G'* > *G"*). In conclusion, API can be considered an appropriate substitute for EY in LFM production, which can benefit human health and offer a new strategy for preparing vegan products.

## Graphical abstract

Amaranth protein has been studied as a promising substitute for unfavorable high cholesterol levels in mayonnaise egg yolk. The alkali extraction/acid precipitation method was conducted at different pHs to promote protein extraction yield. Mayonnaise samples with different amaranth protein isolate/egg yolk powder composition ratios were characterized.

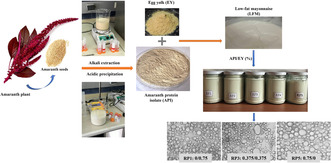

## 1 INTRODUCTION

Mayonnaise is a typical semi‐solid, oil‐in‐water emulsion prepared by mixing oil, egg yolk, vinegar, spices, and additives (Raikos et al., 2020). Egg yolk is an essential ingredient in mayonnaise formulation due to its nutritional and functional properties, like emulsifying and gelling (Laca et al., 2010). During mayonnaise preparation, oil droplets are dispersed in the continuous aqueous phase and tend to flocculate due to the forces of attraction (Ghoush et al., 2008). Here, phospholipids, lipoproteins, and other phosphatides in egg yolk can be absorbed at the oil–water interfaces, forming a viscoelastic film around the oil droplets, reducing surface tension, preventing flocculation/coalescence phenomena, and stabilizing mayonnaise emulsion (Armaforte et al., 2021). However, egg yolk consumption is related to some health issues, such as cardiovascular diseases (due to high cholesterol levels), allergic reactions, and the risk of microbial (*Salmonella enteritidis*) contamination. Furthermore, the demand for healthy food and vegan diets has increased recently (Ali & EL Said, 2020; Eslamian Amiri et al., 2021; Karshenas et al., 2018; Mehdizadeh et al., 2022; Raikos et al., 2020). Therefore, different attempts have been made to develop egg‐free mayonnaise with characteristics similar to those of traditional products (Raikos et al., 2020). However, finding appropriate ingredients to replace egg yolk without disrupting stability, rheology, and sensory perception is challenging (Liu et al., 2018).

Plant proteins could be used as emulsion stabilizers or egg yolk alternatives due to their potential to reduce the surface tension between hydrophilic and hydrophobic components (Nikzade et al., 2012). The use of plant proteins would benefit cholesterol reduction, microbiological stability, and manufacturing costs and draw attention to non‐conventional crops (e.g., pseudocereals) (Nikzade et al., 2012; Riscardo et al., 2003; Suarez & Añón, 2018). Different plant proteins have been used as egg yolk substitutes in mayonnaise formulations. Ozcan et al. (2023) found that egg yolk replacement with aquafaba protein increased the droplet size of mayonnaise, and the emulsion stability decreased after a 50% substitution ratio. The density of mayonnaise increased with increasing aquafaba substitution, while textural parameters (firmness, consistency, cohesiveness) decreased. Mirsadeghi Darabi et al. (2022) observed higher stability and stiffness for mayonnaise emulsions containing clover sprout protein (CSP). CSP replacement increased the pH and decreased the lightness and acidity of mayonnaise. In another study, using different legumin proteins (faba bean, chickpea, and yellow split lentils) led to the production of stable mayonnaise samples with sensory properties comparable to egg yolks containing traditional mayonnaise. However, the substitution caused a reduction in lightness and an increment in the droplet size of mayonnaise emulsions (Armaforte et al., 2021). Ouraji et al. (2020) reported that using faba bean protein isolate as an egg yolk substitute decreased the reduced‐fat mayonnaise lightness and droplet size distribution. Furthermore, the mayonnaise with a fababean protein substitution ratio of 50% had the smallest droplet size among all the samples, but all samples were highly stable.

Amaranth is a pseudocereal with a short life cycle that needs little water (Constantino & Garcia‐Rojas, 2020). It is also highly resistant to poor soils, easily adapted to altitudes, and climate‐resilient (Zhang et al., 2023). One of the interesting features of amaranth is its protein content (14%–18%), which is higher than that of the most common grains (Ventureira et al., 2012). Amaranth is gluten‐free, so it is suitable for people with gluten allergies (Zhang et al., 2023). The protein fractions of amaranth are albumin (~65%), globulin (~17%), prolamin (~11%), and glutelin (~7%) (Janssen et al., 2017). It also has a well‐balanced amino acid composition (high in lysine, tryptophan, and sulfur‐containing amino acid content), high bioavailability, and the potential to release antihypertensive, antioxidant, and antithrombotic peptides after consumption, which indicates its nutritional value (Cortez‐Trejo et al., 2021; Ventureira et al., 2012). Moreover, the essential amino acid content of amaranth protein is relatively close to the amount recommended by the FAO/WHO (Zhang et al., 2023). According to the WHO, amaranth is the second most valuable nutrient in the world, and it can supply the nutrient requirements recommended by the WHO for individuals (Hosseintabar‐Ghasemabad et al., 2022). Also, amaranth seeds have been listed by the FAO as one of the most promising future crops for the world population (Jan et al., 2023). The main purpose of amaranth application in the food industry is in the form of whole grain flour in the preparation of gluten‐free products such as bread (Alsaiqali et al., 2023; Tareh et al., 2022; Yeşil & Levent, 2022), cookies (Chauhan et al., 2016), and pasta (Lux et al., 2023; Schoenlechner et al., 2010). However, it was reported that it should be used with wheat or other cereal flour due to the lack of gliadin in amaranth flour and an inability to form a gluten network. Otherwise, some formulation adjustments (such as using suitable additives and enough water) are needed (Azizi & Azizi, 2023). Amaranth seed is a superfood rich in nutrients such as essential amino acids, vitamins (e.g., E, B_6_, C, riboflavin), minerals (iron, calcium, zinc, etc.), and dietary fiber (Caselato‐Sousa & Amaya‐Farfan, 2012; Ruth et al., 2021). Its flour has been used as a supplement flour in cereal‐based products. Studies showed an increase in the nutritional value of amaranth‐wheat‐based bread (Cotovanu et al., 2023; Emire & Arega, 2012). Amaranth flour was used to produce gluten‐free beer‐like products (Manassero et al., 2020; Meo et al., 2011) and functional beverages (Aderibigbe et al., 2022). Moreover, amaranth flour was tested as a raw material for producing edible films (Tapia‐Bl'acido et al., 2013; Tapia‐Blácido et al., 2005). However, there are few studies on the use of amaranth protein in the food industry. The efficient use of amaranth protein in food formulation highly depends on its functional properties, such as solubility, emulsifying/forming capacity, and water/oil absorption capacity. The type of proteins, their structural properties, and the purity of extracted proteins affect these properties (Das et al., 2021). Protein's emulsifying capacity is closely related to solubility, and both are strongly influenced by protein structural expansion and flexibility of peptide chains caused by changes in protein surface charge due to changes in pH conditions (Shevkani et al., 2014; Ventureira et al., 2010). Besides the influence of the pH of the food system, protein functionality may depend on the interactions with other ingredients in the food formulation and the textural attributes of the target product (Gürbüz et al., 2018).

To the best of our knowledge, there are no reports of using amaranth protein as an egg yolk replacement in low‐fat mayonnaise preparation yet. Therefore, this study aimed to evaluate the properties of amaranth protein isolate (API) and investigate its application as an egg yolk replacer in low‐fat mayonnaise.

## 2 MATERIALS AND METHODS

### 2.1 Materials

Amaranth seeds were supplied by Aplex‐Peru (Lima, Peru). Mayonnaise ingredients: egg yolk powder (Ovodan), pre‐gelatinized waxy maize starch (Acetylated distarch adipate, PregefloCH20, Roquette), xanthan gum (Fufeng), guar gum (Rama Gum Industries India Limited), citric acid, potassium sorbate, and sodium benzoate (Foodchem International Corporation), mustard powder (G. S. Dunn Ltd.), vinegar (11%), sugar, salt, and soybean oil were provided by the R&D department of Behrouz Nik Food Industries. All the chemicals used in the experiments were of analytical grade.

### 2.2 Preparation of amaranth protein isolate (API)

Protein isolate was obtained using an alkali extraction/acidic precipitation methodology described by Constantino and Garcia‐Rojas (2022) with slight modifications. Amaranth seeds were ground to pass a 100‐mesh sieve and defatted by extraction with n‐hexane at a solid/solvent ratio of 1:5 (w/v) under continuous stirring for 8 h at room temperature. The solvent was separated, and the dried residue was suspended in distilled water at a flour/water ratio of 1:8 (w/v). The suspension pH was adjusted to 10 using 1 N NaOH. After stirring for 1 h, the suspension was centrifuged (UNIVERSAL 320R, Hettich, Vlotho, Germany) (4000 × *g*, 15 min) at 20°C. The supernatant was collected, and the procedure was repeated twice. The pH of the collected supernatants was adjusted to 5 with 1 N HCl, and the solution was centrifuged (4000 × *g*, 15 min) at 4°C. The precipitate was resuspended in water, neutralized, and centrifuged. The obtained sediment was dried in an air‐circulation oven at 35°C for nearly 45 h. The dried protein isolate was ground and screened through a 100‐mesh sieve (Karimi et al., 2022).

### 2.3 Properties and evaluation of amaranth protein isolate

#### 2.3.1 Color measurement

The color parameters (L*, a*, and b*) of API and mayonnaise samples were measured using the Hunter lab color measurement system (Reston VA Color Flex No45/0).

#### 2.3.2 Zeta potential (ζ)

The zeta potential of API‐diluted solutions (0.5 g/L) at different pH values of 2, 4, 6, 8, and 10 was measured using a Zetasizer Nano ZS instrument (Malvern Instruments Ltd.) at 25°C.

#### 2.3.3 Protein solubility

2% (w/v) isolate dispersions were prepared at different pH values (2, 4, 6, 8, and 10) using 0.1 N HCl or 0.1 N NaOH. The dispersions were stirred on a magnetic stirrer for 30 min and centrifuged for 15 min at 3000 × *g* (Liang & Tang, 2013). The soluble proteins were determined by the Biuret method (Pinckney, 1961) using different concentrations of BSA as the standard. Protein solubility was calculated using the following equation:

$$\text{Solubility}\;\left(\%\right)=\frac{C_{1}}{C_{2}}\times 100$$

where *C*_1_ and *C*_2_ are the concentrations of proteins in the samples before centrifugation and the supernatant after centrifugation, respectively.

#### 2.3.4 Water absorption capacity (WAC) and oil absorption capacity (OAC)

One gram of API was weighed into pre‐weighed centrifuge tubes and mixed with 10 mL of distilled water or soybean oil for 45 min, followed by centrifugation (4000 × *g*, 10 min) at 25°C. The remaining water or oil was separated from the sediment by inverting the tube at 45° for 10 min (Tomotake et al., 2002). Absorption capacities were expressed as the gram of water or oil remaining per gram of API samples and calculated as follows:

$$\mathrm{WAC}\;\text{or}\;\mathrm{OAC}\;\left(\%\right)=\frac{W_{2}-W_{1}}{W_{0}}$$

where *W*_0_ is the sample weight (g), *W*_1_ is the weight of the tube plus the dry sample (g), and *W*_2_ is the weight of the tube and the sediment (g).

#### 2.3.5 Emulsifying properties

Protein suspensions of 1% (w/v) were prepared, and their pH was adjusted to the desired values (2, 4, 6, 8, and 10) with either 0.1 N NaOH or 0.1 N HCl. 10 mL of suspension and 10 mL of soybean oil were homogenized using a digital homogenizer (IKA‐T18, ULTRA‐TURRAX, Staufen, Germany) for 3 min at 12000 rpm. Then, for emulsifying capacity measurement, the prepared emulsion was centrifuged (3000 × *g*, 5 min), and the volume of the whole content (*V*_1_) and the emulsified fraction (*V*_2_) were recorded. To determine emulsion stability, the emulsions prepared as described were heated (80°C, 30 min), cooled to room temperature, and centrifuged (3000 × *g*, 5 min). The remaining emulsified fraction volume (*V*_3_) was measured (Marcone & Kakuda, 1999). Emulsifying capacity (EC) and emulsion stability (ES) were calculated as follows:

$$\mathrm{EC}\;\left(\%\right)=\frac{V_{2}}{V_{1}}\times 100$$

$$\mathrm{ES}\;\left(\%\right)=\frac{V_{3}}{V_{1}}\times 100$$

### 2.4 Low‐fat mayonnaise preparation

Samples were prepared according to Behrouz Nik Company's commercial low‐fat mayonnaise (LFM) production procedure using API in combination with other ingredients. Water, egg yolk (EY) powder (0%–0.75%), API (0%–0.75%), pre‐gelatinized modified starch (E1422 = 3%), and gums (xanthan = 0.2%; guar = 0.05%) were mixed in the vacuum mixer‐homogenizer (VMH‐Lab, Arkan Felez, Qazvin, Iran), followed by the addition of other powder ingredients, including salt (1.8%), sugar (4.5%), mustard powder (0.5%), citric acid (0.1%), potassium sorbate (0.01%), and sodium benzoate (0.06%). After complete mixing, the oil (30%) was gradually added to the mixer‐homogenizer. Vinegar (4.5%) was added at the end of the process. The overall emulsification procedure took 10 min. Five mayonnaise samples were prepared with different API/EY composition ratios (%) as independent variables, including RP1 as Control: 0/0.75, RP2: 0.25/0.5, RP3: 0.375/0.375, RP4: 0.5/0.25, and RP5: 0.75/0.

### 2.5 Mayonnaise characterization

#### 2.5.1 Droplet size measurement

Mayonnaise samples were diluted with 20 mL of 1% sodium dodecyl sulfate solution and analyzed by Mastersizer 2000 (Malvern Instrument Ltd.) using a laser beam with *λ* = 634 nm at 25°C. A refractive index value of 1.47 was used for the disperse phase (soybean oil) and 1.33 for the continuous phase (water). Droplet size measurements were reported as the volume‐weighted mean diameter (*d*_4,3_, μm), surface‐weight mean diameter (*d*_*3,2*_, μm), and span (span is a measure of the width of a distribution) (Ozcan et al., 2023).

$$d_{3,2}=\Sigma_{i}\;n_{i}\;{d_{i}}^{3}/\Sigma_{i}\;n_{i}\;{d_{i}}^{2}$$

And

$$d_{4,3}=\Sigma_{i}\;n_{i}\;{d_{i}}^{4}/\Sigma_{i}\;n_{i}\;{d_{i}}^{3}$$

where *n*_*i*_ is the number of droplets with the same diameter and *d*_*i*_ is the droplet size.

#### 2.5.2 Optical microscopy

A light microscope (Olympus, BX51M) was used to observe the microstructure of mayonnaise samples. A drop of each sample was placed on a microscope slide covered with a cover slip glass, and images were obtained with a magnification of 100×.

#### 2.5.3 Emulsion stability

20 g of the sample (*F*_0_) was transferred into a pre‐weighed 50 mL centrifuge tube, then heated at 80°C for 30 min and centrifuged (4000 × *g*, 15 min) to remove the top oil layer. The weight of the precipitated fraction (*F*_1_) was measured (Mun et al., 2009). The emulsion stability was calculated as follows:

$$\text{Emulsion stability}\;\left(\%\right)=\frac{F_{1}}{F_{0}}\times 100$$

#### 2.5.4 Freeze–thaw stability

Samples were stored in a freezer at −18°C for 22 h. After freezing, samples were thawed in a water bath at 30°C for 1 h (Zhang et al., 2017), followed by centrifuging at 4000 × *g* for 15 min. The freeze–thaw cycle was repeated three times, two cycles in a row and the third with a seven‐day interval from the first cycle. Samples were stored at 4°C between the second and third cycles. The freeze–thaw stability of emulsions was measured after each cycle using the emulsion stability equation.

### 2.6 Rheological analysis

#### 2.6.1 Steady‐shear rheology

The rheological measurements were performed using an MCR501 rheometer (Anton Paar, Physica, GmbH, Graz, Austria). Flow properties of mayonnaise samples were determined at 25°C using a parallel plate with a diameter of 25 mm (gap = 1 mm), in the shear rate range of 0.01–1000 s^−1^, fitted to the Cross model (Yousefi & Ako, 2020):

$$\frac{\eta -\eta_{\infty }}{\eta_{0}-\eta_{\infty }}=\frac{1}{1+{\left(\lambda ẏ\right)}^{m}}$$

where *η*_*0*_ is the zero‐shear viscosity (Pa.s); *η*_*∞*_ is the infinite shear viscosity (Pa.s); *λ* is the relaxation time (s) for Newtonian Fluids = 0, and *m* is the shear thinning index, which ranges from 0 (Newtonian) to 1 (infinitely shear thinning).

#### 2.6.2 Dynamic rheology

The linear viscoelastic range (LVR) was determined by applying a strain sweep (0.01%–100%) at 1 Hz. The viscoelastic behavior of mayonnaise samples was then measured under a frequency sweep range of 0.01–100 Hz and a constant strain value of 1% (within the LVR). Viscoelastic parameters of storage modulus (*G′*) and loss modulus (*G"*) were recorded as a function of frequency (Ouraji et al., 2020).

### 2.7 Sensory evaluation

Twenty semi‐trained panelists between the ages of 25 and 50 were selected to evaluate the sensory properties of mayonnaise samples that were prepared with different concentrations of API. The evaluation was conducted after a month of preparation of samples using a 5‐point hedonic scale. The sensory evaluation indices were appearance, color, taste, odor, mouthfeel, and overall acceptability.

### 2.8 Statistical analysis

Instrumental measurements were made in duplicate and others in triplicate. A one‐way ANOVA was applied for means with significant differences (*p* < .05), and post hoc analysis was conducted by applying Tukey's test. All statistical analyses were performed using Minitab 21.2 software. Results were expressed as mean values ± standard deviation (SD).

## 3 RESULTS AND DISCUSSION

### 3.1 Amaranth protein isolate (API)

#### 3.1.1 Color

One of the important factors in evaluating the use of plant‐based proteins in food systems is their color, as it affects the final product's appearance. The color of plant proteins is related to the protein‐phenol interactions that form polymers with more pigmentation. In this study, the API's color parameters were L* = 70.24, a* = 2.86, and b* = 14.16. Compared to the Shevkani et al. (2014) report, our API powder showed a lower L* value, while a* and b* had higher values. Findings suggest that oven drying could cause the Maillard reaction, which produces darker colors due to the reaction between amine compounds and aldehydes (melanoidins).

#### 3.1.2 Zeta potential (*ζ*)

**FIGURE 1:**
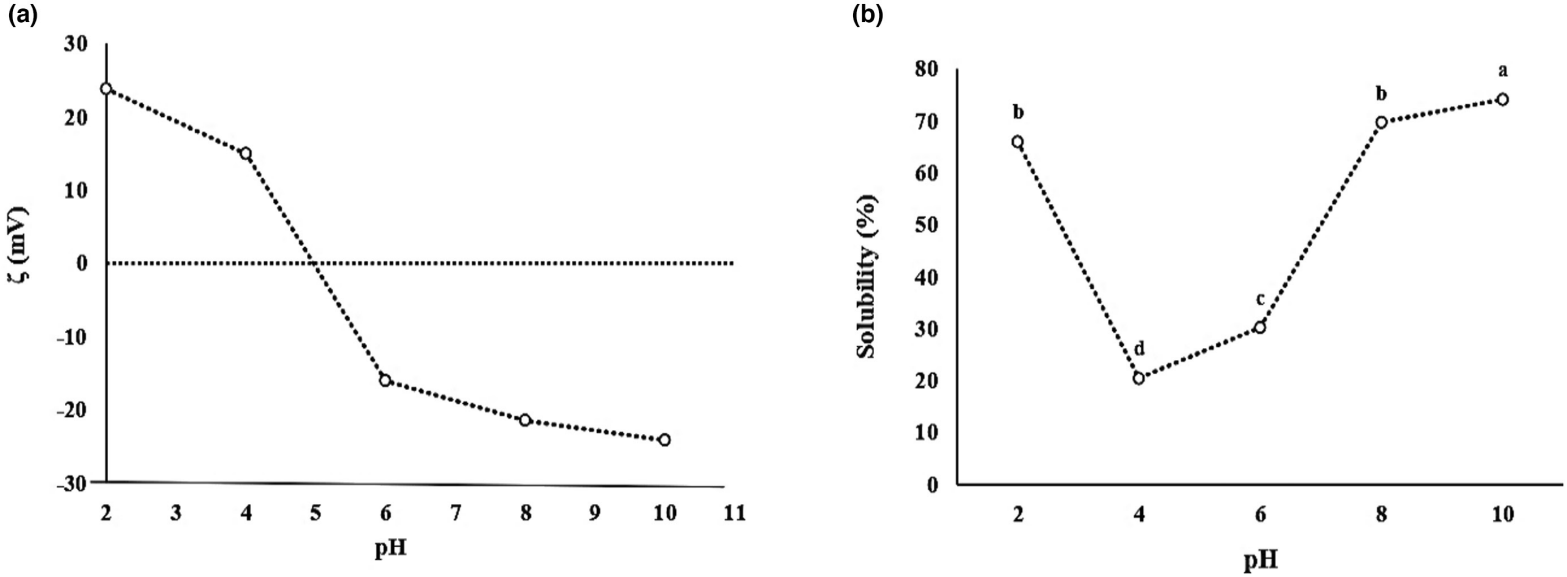
(a) *ζ*‐Potentials of API and (b) API solubility (%) at different pH values. Different letters indicate significant differences (*p* < .05).

Zeta potential indicates the electrical charge around the protein's surface. The *ζ* importance is due to its influence on the protein's functional properties, such as solubility, emulsifying, and foaming (Shevkani et al., 2014). Figure 1a shows the gradual change of the *ζ* value from +23.82 at pH = 2.0 to −24.15 at pH = 10.0. This trend validates the fact that the electrostatic repulsion pattern between negatively and positively charged proteins might gradually change due to the gradual protonation and deprotonation of the proteins' carboxyl and amino groups (Tang & Sun, 2011). Similar *ζ* profiles with the pH were reported, with *ζ* ranging from 30 mV at pH = 3.0 to −30 mV at pH = 10.0 (Constantino & Garcia‐Rojas, 2020) and from 19.6 mV at pH = 2.0 to −37.5 mV at pH = 10.0 (Figueroa‐González et al., 2022). In our study, the isoelectric point (PI), where *ζ* = 0, was almost pH = 5.0. This result is consistent with the mean PI reported between pH 4 and 6 for different amaranth protein fractions by Constantino and Garcia‐Rojas (2020).

#### 3.1.3 Protein solubility

Protein solubility involves hydrophobic (protein–protein) and hydrophilic (protein‐solvent) interactions with water. It is affected by the protein's polar and non‐polar amino acid compositions, its native or denatured state, and the environment. pH is an important environmental factor that highly affects protein solubility (Ulloa et al., 2017). API showed a typical pH‐dependent U‐shaped protein solubility profile; the high solubility values were obtained at pH <4 and >6 (Figure 1b). The low solubility was at pH = 4.0, near the amaranth protein PI (as *ζ* potential data confirms). At the PI, electrostatic repulsive forces between proteins are not strong enough, leading to protein aggregation and solubility reduction (Premkumar et al., 2022). High values of protein solubility at acidic (pH = 2.0) and alkaline (pH = 10.0) conditions could be attributed to the increase in net positive/negative charges leading to stronger electrostatic repulsions between protein molecules. This results in proteins unfolding from and exposing the buried functional groups in protein molecules, increasing the interaction with water (Suarez & Añón, 2018). Similar pH‐dependent behavior with minimum protein solubility around the PI and maximum protein solubility at both acidic and alkaline pH was reported for other plant proteins such as buckwheat, wheat, corn, flaxseed, pea, chickpea, and lentil (Hassan et al., 2010; Ladjal‐Ettoumi et al., 2016; Premkumar et al., 2022; Tang, 2007; Tirgar et al., 2017). Moreover, API showed significantly (*p* < .05) lower solubility at pH = 2.0 (66.02%) than at pH = 10.0 (74.17%). Lower solubility at pH = 2.0 could be due to the protein structure changing in a way that more hydrophobic groups are exposed (Jiang et al., 2009).

#### 3.1.4 Water absorption capacity and oil absorption capacity

The water‐binding property of a protein is related to its polar amino acid groups, which are the main sites of water–protein interactions (Ghumman et al., 2021). The water absorption capacity of API obtained in this study was 2.38 g/g. Shevkani et al. (2014) reported the water absorption capacity of six different cultivars of amaranth ranging between 1.0 and 3.3 g/g. Proteins with water absorption capacity values between 1.49 g/g and 4.72 g/g have been reported to be better suited for processing viscous foods, which absorb water without the protein dissolving, thus providing thickening and viscosity (Aletor et al., 2002).

Oil absorption capacity describes the efficiency of proteins in binding to oils, resulting from the interaction between the hydrophobic amino acids in proteins and the hydrocarbon molecules of oils (Premkumar et al., 2022). The oil absorption capacity of API was measured to be 4.15 g/g, which lies within the oil absorption capacity range (3.6–6.4 g/g) reported by Shevkani et al. (2014). The differences in protein isolates' water and oil absorption capacities can be attributed to various conformational characteristics (hydrophilic/hydrophobic areas on the protein surface) and purity of the isolates (López et al., 2019). According to our findings, API appears to contain a sufficient amount of nonpolar amino acids that can interact with lipid hydrocarbon chains, making it appropriate to be used as a fat replacer or extender in emulsion foods such as mayonnaise, salad dressings, soups, and sausages.

#### 3.1.5 Emulsifying capacity and emulsion stability

**FIGURE 2:**
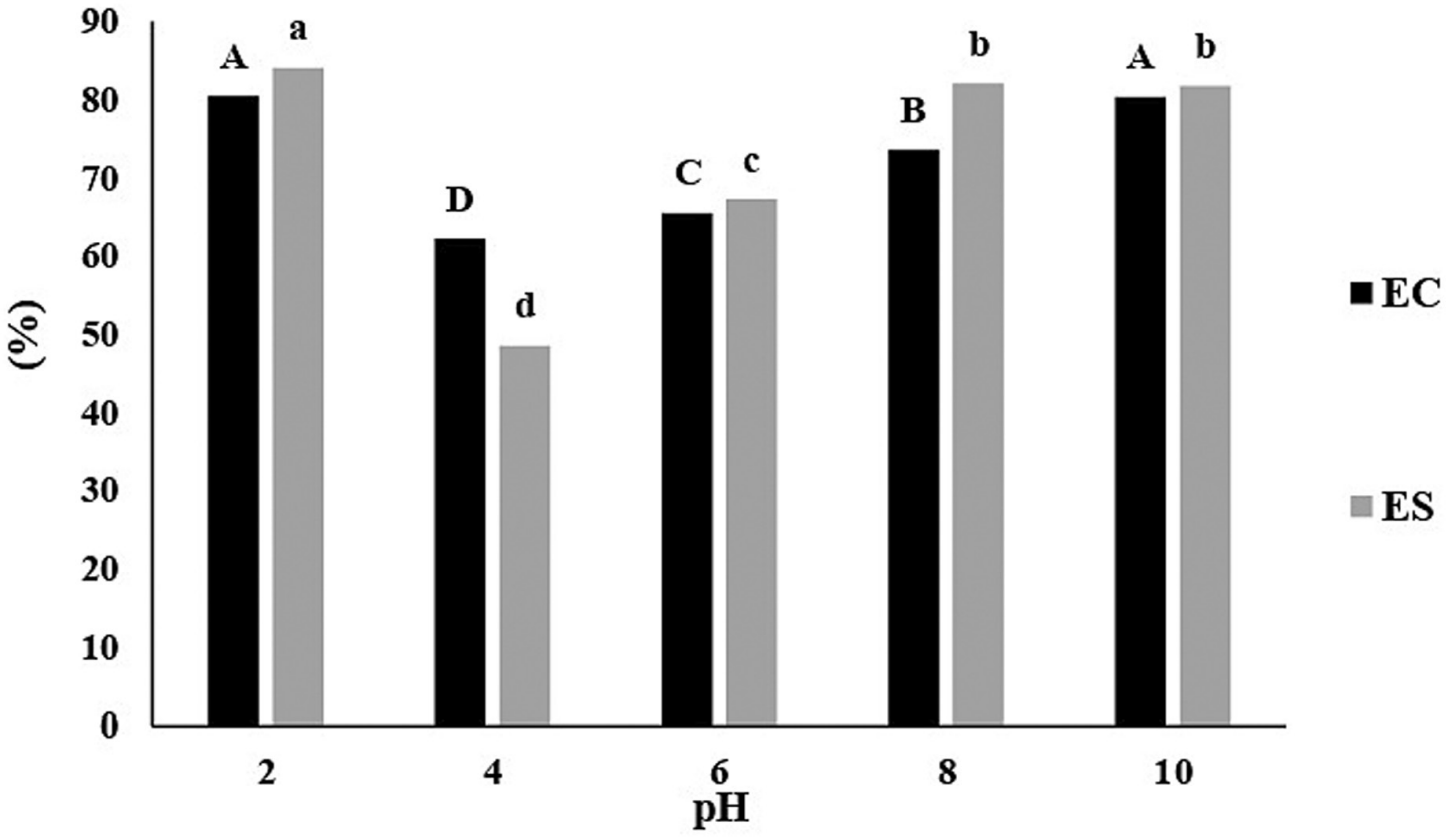
API emulsifying properties: emulsifying capacity (EC) and emulsion stability (ES) at different pH values. Different capital letters (black bars) and different lowercase letters (gray bars) indicate significant differences (*p* < .05).

Emulsifying capacity reflects the amount of oil that a standard amount of protein can emulsify in an oil/water (O/W) or water/oil (W/O) emulsion without separating, while emulsion stability indicates the ability of the protein to keep the emulsion stable over a determined period by preventing flocculation or coalescence of the oil droplets (Tirgar et al., 2017). As shown in Figure 2, API emulsifying properties had a pH‐dependent behavior, with the lowest emulsifying capacity at pH = 4.0 (62.22%), nearly close to the amaranth protein PI. Due to the positive and negative charge balance at PI, the interaction between nearby protein molecules is enhanced, resulting in protein aggregation. This prevents proteins from efficient diffusion at the oil–water interface, resulting in emulsifying capacity reduction (Yuliana et al., 2014). High emulsifying capacity was evident at pH = 2.0 (80.49%) and pH = 10.0 (80.38%). At acidic or alkaline pH, proteins have either net positive or net negative charges, which favor electrostatic repulsion force that helps keep proteins apart, disturbing protein native structure and shifting the equilibrium toward the unfolded form, so the hydrophobic and hydrophilic groups inside the protein are exposed, which in turn enhances the oil and water binding of protein; therefore, emulsifying capacity increases (Jiang et al., 2009; Yuliana et al., 2014). Dias and de Moura Bell (2022) reported a similar relationship between emulsifying capacity and pH for almond protein. In this study, similar results were obtained with emulsion stability as with emulsifying capacity (Figure 2). The lowest and highest significant values (*p* < .05) of emulsion stability were observed at pH = 4.0 (48.51%) and pH = 2.0 (84.06%), respectively. Proteins with higher emulsifying capacity and emulsion stability are desirable for emulsified foods such as salad dressing, mayonnaise, sausages, and meat products.

### 3.2 Mayonnaise

#### 3.2.1 Color

Food color is critical to any food's appearance, as it significantly affects its appeal and indicates food quality (Premkumar et al., 2022). There is no significant difference (*p* > .05) between the lightness index (L*) values of the control sample (RP1) and API‐containing mayonnaise samples (Table 1). Substitution of API showed no significant effect on the mayonnaise redness index (a*) except for the RP3 sample, which was meaningfully higher (*p* < .05) than the other samples. The yellowness index (b*) was significantly (*p* < .05) affected by API replacement. The RP5 showed a higher b* value among all samples. The change in the mayonnaise color parameters due to substitution can be related to the yellowish‐brown color of the API. However, the EY replacement with API had no adverse impact on the mayonnaise color feature.

**TABLE 1:** Color and particle size of low‐fat mayonnaise samples.

Samples	Color parameters^1^			Particle size^2^		
	L*	a*	b*	*d*_3,2_ (μm)	*d*_4,3_ (μm)	Span
RP1	83.63 ± 0.17^a^	0.38 ± 0.04^b^	9.39 ± 0.03^c^	3.86 ± 0.03^c^	8.80 ± 0.07^c^	2.83 ± 0.05^c^
RP2	83.46 ± 0.41^a^	0.32 ± 0.02^b^	9.14 ± 0.05^d^	4.38 ± 0.14^b^	12.87 ± 0.16^b^	4.48 ± 0.18^b^
RP3	83.81 ± 0.06^a^	0.5 ± 0.02^a^	9.21 ± 0.01^d^	5.18 ± 0.11^a^	43.64 ± 0.2^a^	6.15 ± 0.08^a^
RP4	83.84 ± 0.03^a^	0.39 ± 0.01^b^	9.71 ± 0.02^b^	3.96 ± 0.08^c^	9.30 ± 0.14^c^	3.54 ± 0.37^c^
RP5	83.62 ± 0.02^a^	0.32 ± 0.01^b^	10.05 ± 0.02^a^	3.98 ± 0.04^c^	8.91 ± 0.08^c^	3.51 ± 0.01^c^

*Note*: Results are shown as mean ± SD. Different letters in the same column indicate a significant difference between the mean values (*p* < .05).

^1^ Measurement performed in triplicate.

^2^ Measurement performed in duplicate.

#### 3.2.2 Droplet size and microstructure

As shown in Table 1, the RP1 sample had the lowest values of *d*_*3,2*_ and *d*_*4,3*_ among all samples. Low concentrations of API, 0.25% (RP2) and 0.375% (RP3), significantly (*p* < .05) increased the oil droplet size of the mayonnaise emulsion compared with the RP1. However, the oil droplet size decreased with the increase in API concentration (0.5% and 0.75%). No significant differences (*p* > .05) were observed between the RP1, RP4, and RP5 samples. The oil droplet size is an important property due to its effect on emulsions' rheology, stability, storage life, texture, and taste (Yildirim et al., 2016). Large oil droplet size indicates oil droplets' tendency to flocculation/coalescence; hence, a decrease in this characteristic implies higher emulsion stability (Drozłowska et al., 2020). The span parameter shows the particle size distribution, i.e., higher span values suggest that the emulsion contains a broader range of oil droplet size. Generally, emulsions with homogeneous particle size distribution (monodisperse) have been related to better stability (Metri‐Ojeda et al., 2022). The RP3 sample significantly (*p* < .05) showed a higher span value (Table 1). This might be due to the antagonistic effect of egg yolk and API proteins, which reduces their emulsifying ability, resulting in emulsion formation with larger non‐uniform droplets (polydisperse).

**FIGURE 3:**
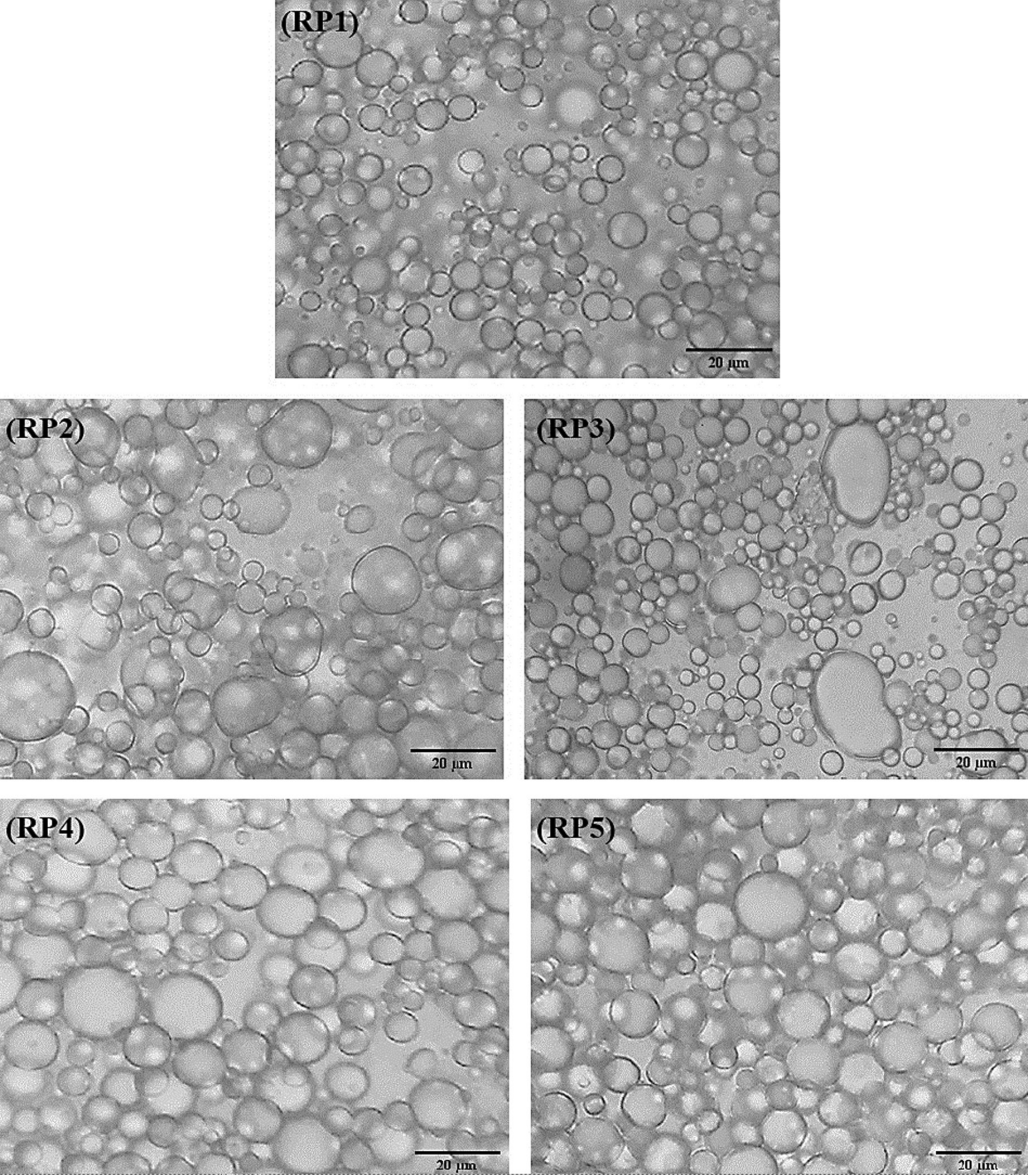
Microstructure images of low‐fat mayonnaise samples for 100× magnification.

Figure 3 shows the microstructure images of LFM samples with different concentrations of API (0%–0.75%) 2 weeks after preparation. Various factors could affect the microstructure of the emulsion, including the type and concentration of emulsifying and stabilizing agents, droplet size, water phase viscosity, and oil content (Laca et al., 2010). An ideal emulsion comprises spherical droplets packed together within the continuous phase (Depree & Savage, 2001). The emulsion of mayonnaise samples RP1, RP4, and RP5 contains round oil droplets trapped in the continuous aqueous phase. The RP1 displayed more monodisperse oil droplets among all samples. Additionally, it can be observed that the RP4 and RP5 samples contained more tightly packed oil droplets than the RP1 sample. The RP3 emulsion demonstrates larger oil droplets and a high degree of polydispersity, with less compacted oil droplets than the other samples. These findings are consistent with our droplet size measurement results.

#### 3.2.3 Emulsion stability and freeze–thaw stability

**FIGURE 4:**
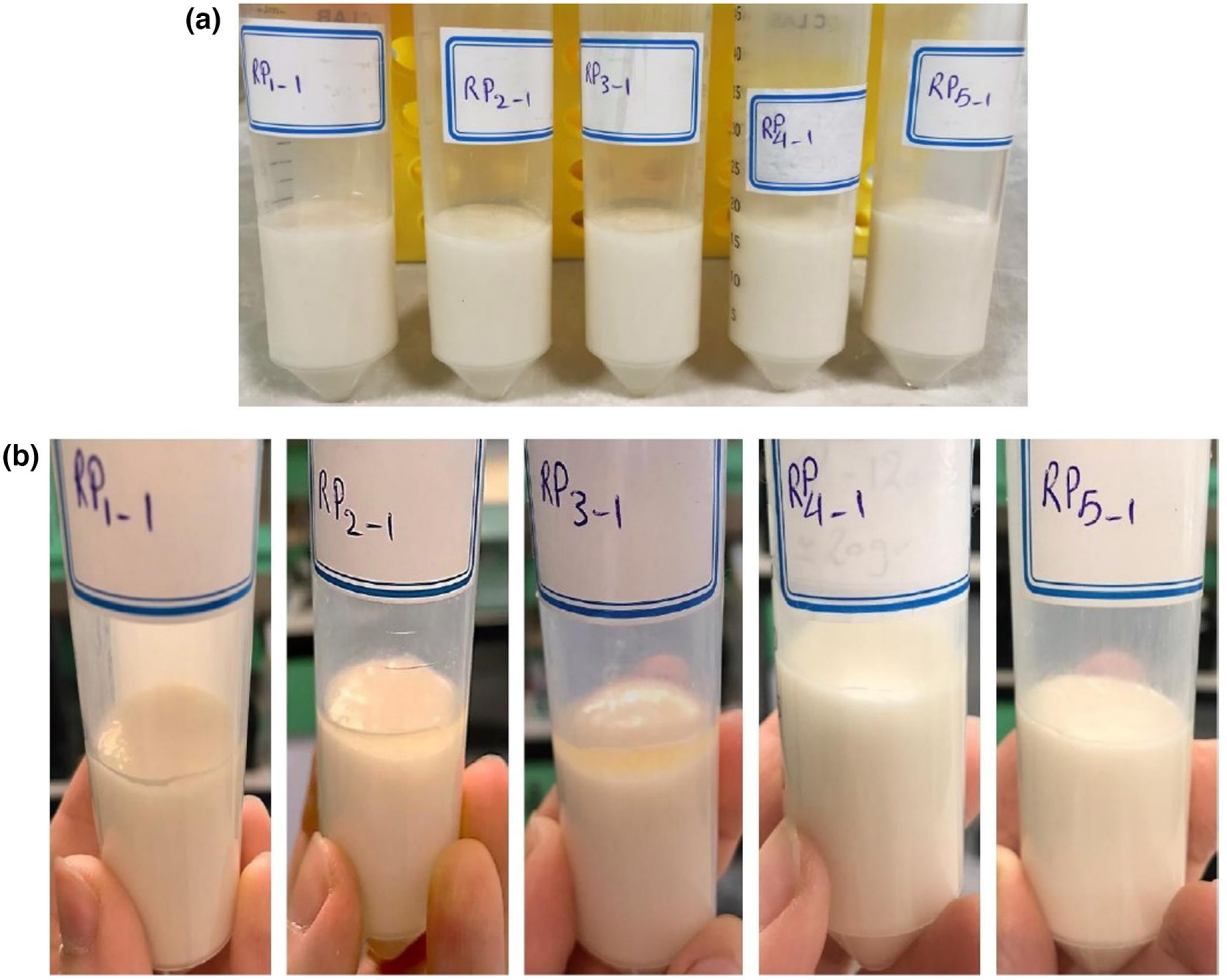
(a) Emulsion stability and (b) freeze–thaw stability of low‐fat mayonnaise samples.

Emulsion stability measurement is critical to the shelf‐life prediction of many commercial products and is associated with the prevention of coalescence/flocculation of oil droplets and creaming (Nikzade et al., 2012). The results of the emulsion stability and freeze–thaw stability of mayonnaise samples prepared with different API substitution ratios are summarized in Table 2. As can be seen, all samples were highly stable during the heating process (80°C, 30 min) (Figure 4a). The emulsifier's efficiency is an important factor that affects the mayonnaise's stability (Depree & Savage, 2001). Our results indicate the high efficiency of egg yolk and API as emulsifiers; they have maintained their emulsifying ability at high temperatures, meaning their protein emulsifier layer around the oil droplets remained intact, preventing phase separation of the emulsion. These results are consistent with those reported by Ouraji et al. (2020), who have used faba bean as an egg substitute at different levels. They reported that all samples were highly stable due to the reduction of oil/water interfacial tension by the protein emulsifier layer, resulting in the oil droplet's flocculation/coalescence restriction. For freeze–thaw stability, in the first cycle, no phase separation was observed for the RP1, RP4, and RP5 samples (Table 2). The RP2 and RP3 samples showed instability, but the amount was negligible (Figure 4b) (stability >95%). In two other cycles, the RP3 sample significantly (*p* < .05) showed the lowest freeze–thaw stability among all samples. The formation of smaller droplets in the emulsion favors their Brownian motion and the subsequent homogeneous distribution, which restricts the gravitational forces and reduces the droplet distance (Rudra et al., 2020). Moreover, tightly packed oil droplets allow stronger interactions between oil droplets (Park et al., 2020). Narrow size distribution and packed oil droplets provide higher viscosities, and high viscosity prevents emulsion instability by restricting droplet mobility (Park et al., 2020; Rudra et al., 2020). The RP3 sample has the largest droplet size with a higher span value, meaning that spaces in the continuous phase are occupied with more water (Figure 3). This would motivate the formation of large, sharp ice crystals during freezing, which might damage the oil membrane (the protein layer around oil droplets), ultimately resulting in phase separation (Bagheri et al., 2018). Mayonnaise samples containing high API concentrations (RP4: 0.5% and RP5: 0.75%) showed high emulsion and freeze–thaw stabilities. This confirms the results of API emulsifying properties. Besides, amaranth protein displayed acceptable water and oil absorption capacities, which could increase emulsion stability by strengthening the interactions between the protein's hydrophilic regions and continuous phase as well as the hydrophobic protein regions with the oil droplets. Huang et al. (2022) concluded that the freeze–thaw stability of mayonnaise emulsions is due to the presence of oil droplets with strong interfacial stabilization against the oil or ice crystals. They also reported that increasing the number of freeze–thaw cycles may increase the droplet size, which causes partial coalescence of oil droplets and emulsion instability.

**TABLE 2:** Emulsion stability and freeze–thaw stability of low‐fat mayonnaise samples.

Samples	Emulsion stability (%)	Freeze–thaw stability (%)		
		Cycle 1	Cycle 2	Cycle 3
RP1	99.96 ± 0.05^a^	100 ± 0.00^a^	99.95 ± 0.01^a^	98.79 ± 0.5^a^
RP2	99.87 ± 0.03^a^	98.92 ± 0.02^b^	98.87 ± 0.17^b^	94.19 ± 0.5^b^
RP3	99.75 ± 0.14^a^	96.29 ± 0.6^c^	92.87 ± 0.19^c^	89.71 ± 0.65^c^
RP4	99.97 ± 0.03^a^	100 ± 0.00^a^	99.98 ± 0.02^a^	99.94 ± 0.02^a^
RP5	100 ± 0.00^a^	100 ± 0.00^a^	99.97 ± 0.03^a^	100 ± 0.00^a^

*Note*: Results are shown as mean values of triplicate ± SD. Different letters in the same column indicate a significant difference between the mean values (*p* < .05).

### 3.3 Rheological properties

#### 3.3.1 Steady‐shear flow behavior

**FIGURE 5:**
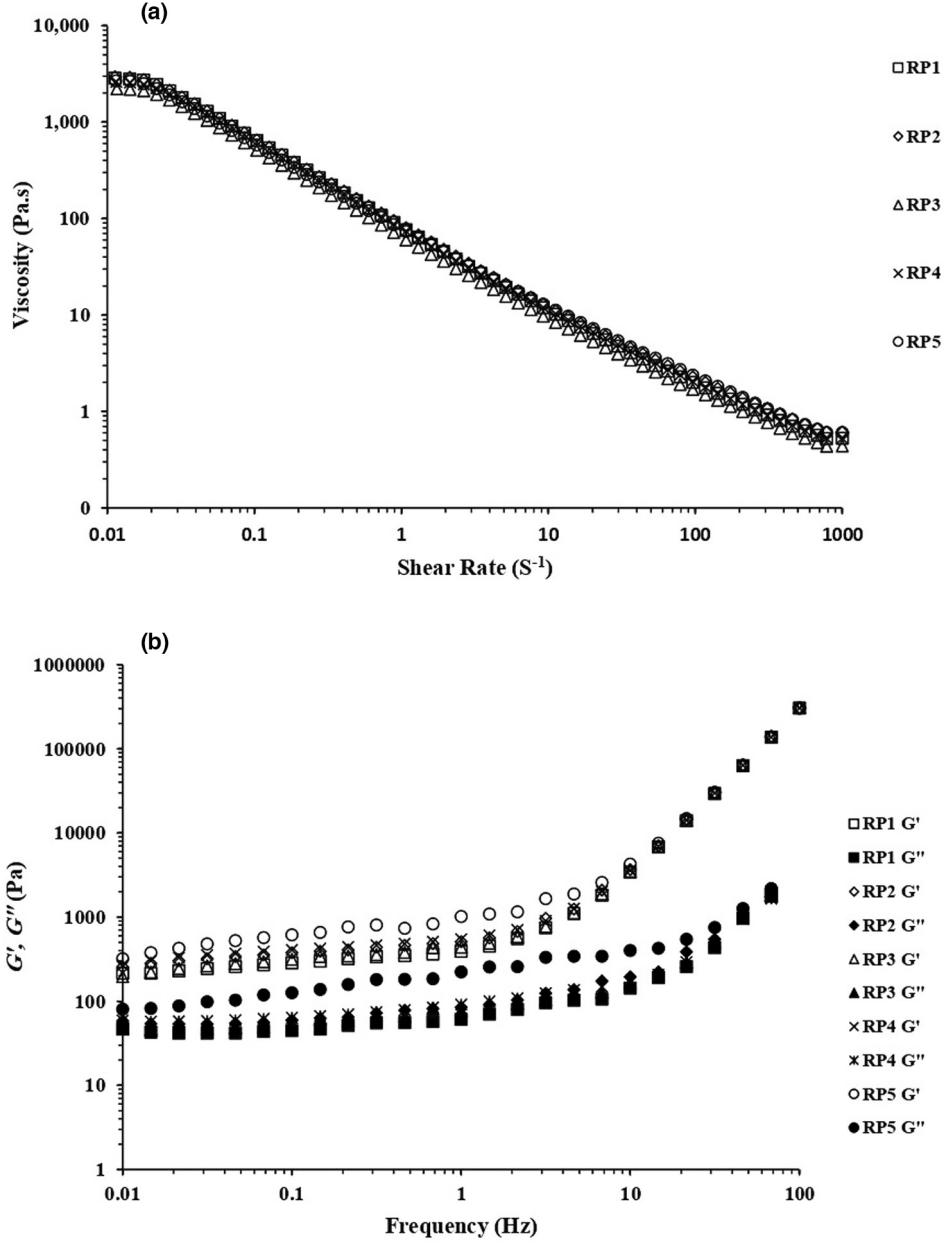
(a) Apparent viscosity (Pa.s) vs. shear rate (s^−1^), (b) storage modulus (*G'*), and loss modulus (*G"*) values of frequency sweeps of low‐fat mayonnaise samples.

The apparent viscosity of mayonnaise samples as a function of shear rate is shown in Figure 5a. All samples showed pseudoplastic shear‐thinning behavior. The reduction in viscosity under shear could be attributed to the structural deformation of droplets and their three‐dimensional network alignment with the flow direction (Bourbon et al., 2010).

In this study, we used the rheological cross model with the higher coefficient of determination (*R* ^2^ = 0.99) to describe the flow behavior of LFM samples (Table 3). This model provides interesting parameters, which help better understand emulsion rheological characteristics.

**TABLE 3:** Flow behavior parameters of the rheological cross model of low‐fat mayonnaise samples.

Samples	*ɳ*_*0*_ (Pa.s)	*ɳ*_*∞*_ (Pa.s)	*λ* (s)	*m*	*R* ^*2*^
RP1	12,939 ± 1.4^c^	0.31 ± 0.02^a^	145.94 ± 0.26^d^	0.88 ± 0.01^a^	0.99
RP2	12680.5 ± 2.1^d^	0.33 ± 0.01^a^	172.91 ± 0.12^c^	0.86 ± 0.02^a^	0.99
RP3	11,633 ± 1.4^e^	0.25 ± 0.01^a^	133.54 ± 0.14^e^	0.87 ± 0.01^a^	0.99
RP4	16,038 ± 1.4^b^	0.29 ± 0.02^a^	179.20 ± 0.4^b^	0.86 ± 0.01^a^	0.99
RP5	17016.5 ± 2.1^a^	0.3 ± 0.02^a^	198.66 ± 0.63^a^	0.82 ± 0.01^a^	0.99

*Note*: Results are shown as mean values of duplicate ± SD. Different letters in the same column indicate a significant difference between the mean values (*p* < .05). *η*_*0*_ is the zero‐shear viscosity, *η*_*∞*_ is the infinite shear viscosity, *λ* is the relaxation time, *m* is the flow behavior index, and *R* ^2^ is the coefficient of determination.

Zero‐shear viscosity (*η*_*0*_) represents the emulsion's microstructural changes during storage. The higher *η*_*0*_, the higher the stability of the mayonnaise emulsion against creaming or droplet accumulation over storage time (Razavi et al., 2014). The RP3 and RP5 samples showed the significantly (*p* < .05) lowest and highest values of *η*_*0*_, respectively (Table 3). The lowest value of *ɳ*_*0*_ can be attributed to the antagonist relationship between egg yolk and API proteins, forming a weaker emulsifier layer and increasing interfacial tension (Wan et al., 2014; Zhang et al., 2022). Also, large and less uniform oil droplet size could be associated with lower *η*_*0*_ (Shariful et al., 2018). Compared to the RP1, the RP4 and RP5 samples showed a significantly higher value of *η*_*0*_ (*p* < .05). The higher value of *η*_*0*_ in the presence of high API concentrations (0.5% and 0.75%) indicates the establishment of greater intermolecular interactions between the mayonnaise emulsion particles (Bourbon et al., 2010).

The infinite shear viscosity (*η*_∞_) demonstrates the consistency of the food fluids, such as emulsions, under processing operations like mixing, pumping, or any fast movements. In short, higher *η*_*∞*_ values require more energy for food processing (Razavi et al., 2014). As can be seen in Table 3, there are no significant differences (*p* > .05) between mayonnaise samples. This indicates that the samples prepared with API substitution need the same amount of energy as the control sample for processing. Furthermore, the rheological properties could affect the emulsion's sensory and textural characteristics. The high viscosity of the emulsions (*η*_*0*_ and *η*_*∞*_) indicates a more pleasant creaminess and mouthfeel.

The relaxation time (*λ*) reflects the average time required for macromolecules to return to a steady state in the shearing medium. It is the opposite of the critical shear rate, which corresponds to the transition of emulsion behavior from Newtonian to non‐Newtonian (Razavi et al., 2014). Compared to RP1, the value of *λ* increased significantly (*p* < .05) for all samples prepared with the API, except for RP3, which was considerably (*p* < .05) lower than other samples. An increase in *λ* means long‐term stability of the emulsion structure, which indicates that more time is needed to replace new entanglements with those disrupted through imposed external deformations (Razavi et al., 2014).

The flow behavior index (*m*) of the cross model indicates the shear‐thinning characteristics of an emulsion. Samples with a higher *m* value (much higher than 0 but less than 1) represent more pseudoplasticity (Bourbon et al., 2010). The flow behavior index (*m*) of LFM samples ranged between 0.82 (RP5) and 0.88 (RP1), which is close to 1, so it can be concluded that they have nearly high pseudoplastic behavior. Moreover, no significant difference (*p* > .05) was found between the samples (Table 3).

#### 3.3.2 Viscoelastic properties

The frequency sweep test is the most common oscillatory test to study the viscoelastic properties of emulsions like mayonnaise. Figure 5b depicts the effect of egg yolk replacement levels with API on *G'* and *G"* in the frequency sweep test. For all low‐fat mayonnaise samples, *G'* was greater than *G"* in the entire frequency range, demonstrating the elastic‐dominant behavior of all samples and suggesting that all have a weak gel structure. Similar trends were also reported previously (Jing et al., 2023; Ouraji et al., 2020). Also, *G'* and *G"* values increased with an increase in API concentration. In low frequencies, the RP1 and RP5 samples showed the lowest and highest storage modulus values (*G'*), respectively. The high *G'* value is related to droplet size and how compact the droplets are. Smaller and more highly packed oil droplets will lead to the formation of a highly viscous and more solid‐like structure that is more resistant to deformation. This shows that high stress is needed to make the emulsion start to flow; in other words, the emulsion will have less pseudoplastic behavior (Jadhav et al., 2022; Thaiudom & Khantarat, 2011). As the flow behavior results showed, the RP5 sample had the lowest *m* value among the other samples. Furthermore, the RP5 sample showed the slightest changes in storage modulus value with the frequency augments compared to other samples. This may have been caused by the strong link between the particles, which results in increased relaxation time (*λ*) (Jadhav et al., 2022). All samples showed almost equal *G'* and *G"* values at high frequencies, indicating their similar viscoelastic behavior (Figure 5b).

### 3.4 Sensory evaluation

Table 4 presents the sensory evaluation scores of the LFM samples; no significant difference (*p* > .05) was observed between the attributes. However, API substitution reduced the appearance score, possibly due to the reduction in the L* value of mayonnaise. Samples with 0.25% and 0.375% API (RP2 and RP3 samples, respectively) got higher scores in color. This could be attributed to the improvement in the creamy color of LFM. However, the color score decreased with increasing API concentration, so the lowest score values were found at 0.75% API (RP5 sample). The highest score for the odor and taste attributes belonged to the RP3 sample, which, according to panelists, was due to its mild sour aroma and flavor. Mouthfeel was determined by pressing a certain amount of mayonnaise with the tongue against the palate, indicating its creaminess and soft texture (Stern et al., 2007). The RP5 sample scored the highest mouthfeel quality among all LFM samples. Rudra et al. (2020) reported higher mouthfeel scores for mayonnaise samples with higher viscosities. They stated that emulsions with high viscosity can be perceived as creamy and are preferred over other emulsions. In our study, the RP5 sample had the highest viscosity, *ɳ*_*0*_, and *ɳ*_∞_ (Table 3), which could be the reason for its high mouthfeel score. The overall acceptability of LFM samples was assessed by appearance, color, taste, odor, and mouthfeel to predict consumer acceptance of the final product. According to the sensory analysis, the RP3 sample with 0.375% API obtained the highest overall acceptability.

**TABLE 4:** Sensory scores of low‐fat mayonnaise samples.

Samples	Appearance	Color	Odor	Taste	Mouthfeel	Overall acceptability
RP1	4.45 ± 0.82^a^	3.95 ± 1.02^a^	4.05 ± 1.09^a^	4.15 ± 0.81^a^	4.3 ± 1.03^a^	4.1 ± 0.78^a^
RP2	4.1 ± 0.55^a^	4.1 ± 0.96^a^	4.1 ± 0.71^a^	4.2 ± 0.69^a^	4.2 ± 0.89^a^	4.1 ± 0.72^a^
RP3	4.15 ± 0.67^a^	4.2 ± 0.95^a^	4.14 ± 0.74^a^	4.3 ± 0.73^a^	4.2 ± 1.02^a^	4.25 ± 1.07^a^
RP4	3.60 ± 0.28^a^	3.85 ± 0.81^a^	4.1 ± 0.71^a^	3.75 ± 1.02^a^	4.35 ± 0.67^a^	4.05 ± 0.79^a^
RP5	3.45 ± 0.49^a^	3.8 ± 0.89^a^	4.00 ± 1.02^a^	3.7 ± 1.26^a^	4.4 ± 0.82^a^	3.9 ± 0.78^a^

*Note*: Different letters in the same column indicate a significant difference between the mean values (*p* < .05).

## 4 CONCLUSION

The influence of pH shifting on API properties showed higher solubility and *ζ*‐ potential values at pH = 10.0, while higher emulsifying capacity and emulsion stability values were found at pH = 2.0. Acidic pH has altered the protein structure in a way that results in better interfacial penetration, stronger interfacial interactions, and greater emulsion stability. API as egg yolk substitution had no significant (*p* > .05) impact on L* and a* values and the emulsion stability of LFM samples. Low API concentrations (0.25% and 0.375%) increased the droplet size and decreased freeze–thaw stability significantly (*p* < .05). The larger oil droplet can be attributed to the antagonistic effects of API and egg yolk proteins. Reversely, high API concentrations (0.5% and 0.75%) showed no remarkable (*p* > .05) impact on droplet size and its homogeneity and had better FTS than the RP1 sample. All mayonnaise samples showed pseudoplastic shear‐thinning behavior, which was fitted to the Cross rheological model (*R* ^2^ = 0.99). The highest and lowest significant values (*p* < .05) of *ɳ*_0_ belonged to the RP5 and RP3 samples, respectively. There were no considerable differences (*p* > .05) for the parameters *ɳ*_∞_ and *m*. The *G'* value of mayonnaise samples was higher than *G"* during the frequency test, indicating their weak gel structure. The LFM samples did not differ significantly (*p* > .05) in sensory characteristics, yet the sample with 0.375% API (RP3 sample) had the highest overall acceptability score. These results suggest that LFM prepared with high API concentrations (0.5% and 0.75%) could be a healthy alternative to traditional LMF, which offers a promising market to attract plant‐based diet lovers.

## AUTHOR CONTRIBUTIONS

**Sahar Mohammadi:** Data curation (equal); investigation (equal); methodology (equal); writing – original draft (equal). **Mazdak Alimi:** Conceptualization (equal); project administration (equal); supervision (equal); validation (equal); writing – review and editing (equal). **Seyed‐Ahmad Shahidi:** Conceptualization (equal); supervision (equal); validation (equal); writing – review and editing (equal). **Shirin Shokoohi:** Supervision (equal); writing – review and editing (equal).

## FUNDING INFORMATION

The authors declare that no funds, grants, or other support were received during the preparation of this manuscript.

## CONFLICT OF INTEREST STATEMENT

Sahar Mohammadi reports that equipment, drugs, or supplies were provided by the R&D department of Behrouz Nik Food Industries. The authors declare that the study does not involve human or animal testing, and they do not have any conflicts of interest.

## ETHICS STATEMENT

The authors will follow the ethical responsibilities of authors and COPE rules. On behalf of all co‐authors, I believe the participants are giving informed consent to participate in this study.

## CONSENT FOR PUBLICATION

All the authors give their consent for the submitted manuscript to be published in *Food Science & Nutrition*.

## Data Availability

All data are presented in the manuscript.
